# Clinical characteristics and host immunity responses of SARS-CoV-2 Omicron variant BA.2 with deletion of ORF7a, ORF7b and ORF8

**DOI:** 10.1186/s12985-023-02066-3

**Published:** 2023-05-29

**Authors:** Zhizhong Tang, Pei Yu, Qianfang Guo, Mingxiao Chen, Yu Lei, Lei Zhou, Weikang Mai, Lu Chen, Min Deng, Weiya Kong, Chuanying Niu, Xiaoli Xiong, Wenrui Li, Chunbo Chen, Changchun Lai, Qian Wang, Baisheng Li, Tianxing Ji

**Affiliations:** 1 Urology Surgery Department, Maoming People’s Hospital, Maoming, 525000 People’s Republic of China; 2 Clinical Laboratory Medicine Department, The Second Affiliated Hospital of Guangzhou Medical University, Guangzhou, 510260 People’s Republic of China; 3 Guangdong Provincial Key Laboratory of Pathogen Detection for Emerging Infectious Disease Response, Institute of Microbiology, Guangdong Provincial Center for Disease Control and Prevention, Guangdong, 511430 People’s Republic of China; 4 Department Of Pathology Laboratory, Maoming People’s Hospital, Maoming, 525000 People’s Republic of China; 5 State Key Laboratory of Respiratory Disease, CAS Key Laboratory of Regenerative Biology, Guangdong Provincial Key Laboratory of Stem Cell and Regenerative Medicine, Guangdong Provincial Key Laboratory of Biocomputing, Guangzhou Institutes of Biomedicine and Health, Chinese Academy of Sciences, Guangzhou, 510535 People’s Republic of China; 6 Bioland Laboratory (Guangzhou Regenerative Medicine and Health-Guangdong Laboratory), Guangzhou, 510005 People’s Republic of China; 7 Clinical Laboratory Medicine Department, Dongguan Ninth People’s Hospital, Dongguan, 523016 People’s Republic of China; 8 Clinical Laboratory Medicine Department, Maoming People’s Hospital, Maoming, 525000 People’s Republic of China; 9 State Key Laboratory of Respiratory Disease, Guangzhou Institute of Respiratory Health, The First Affiliated Hospital of Guangzhou Medical University, Guangzhou, 510120 People’s Republic of China; 10 Intensive Care Unit Department, Maoming People’s Hospital, Maoming, 525000 People’s Republic of China; 11 Guangzhou Key Laboratory for Clinical Rapid Diagnosis and Early Warning of Infectious Diseases, KingMed School of Laboratory Medicine, Guangzhou Medical University, Guangzhou, 511495 People’s Republic of China

**Keywords:** ORF7a, ORF7b, ORF8, Cellular immunity, Humoral immunity, Cytokines

## Abstract

**Background:**

The pathogenicity and virulence of the Omicron strain have weakened significantly pathogenesis of Omicron variants. Accumulating data indicated accessory proteins play crucial roles in host immune evasion and virus pathogenesis of SARS-CoV-2. Therefore, the impact of simultaneous deletion of accessory protein ORF7a, ORF7b and ORF8 on the clinical characteristics and specific immunity in Omicron breakthrough infected patients (BIPs) need to be verified.

**Methods:**

Herein, plasma cytokines were identified using a commercial Multi-cytokine detection kit. Enzyme-linked immunosorbent assay and pseudovirus neutralization assays were utilized to determine the titers of SARS-CoV-2 specific binding antibodies and neutralizing antibodies, respectively. In addition, an enzyme-linked immunospot assay was used to quantify SARS-CoV-2 specific T cells and memory B cells.

**Results:**

A local COVID-19 outbreak was caused by the Omicron BA.2 variant, which featured a deletion of 871 base pairs (∆871 BA.2), resulting in the removal of ORF7a, ORF7b, and ORF8. We found that hospitalized patients with ∆871 BA.2 had significantly shorter hospital stays than those with wild-type (WT) BA.2. Plasma cytokine levels in both ∆871 BA.2 and WT BA.2 patients were within the normal range of reference, and there was no notable difference in the titers of SARS-CoV-2 ancestor or Omicron-specific binding IgG antibodies, neutralizing antibody titers, effector T cells, and memory B cells frequencies between ∆871 BA.2 and WT BA.2 infected adult patients. However, antibody titers in ∆871 BA.2 infected adolescents were higher than in adults.

**Conclusions:**

The simultaneous deletion of ORF7a, ORF7b, and ORF8 facilitates the rapid clearance of the BA.2 variant, without impacting cytokine levels or affecting SARS-CoV-2 specific humoral and cellular immunity in Omicron-infected individuals.

**Supplementary Information:**

The online version contains supplementary material available at 10.1186/s12985-023-02066-3.

## Background

The COVID-19 pandemic, caused by severe acute respiratory syndrome coronavirus 2 (SARS-CoV-2) infection, continues to be a significant public health emergency worldwide [1]. The emergence of SARS-CoV-2 variants of concern (VOC) during the pandemic has contributed to the ongoing spread of the virus in humans, despite widespread vaccination efforts, and poses significant challenges for pandemic control due to increased infectivity, transmission, and ability to evade immunity [2]. Considerable attention has been directed towards the spike protein, which plays a crucial role in the entry of the virus into the host cell through its interaction with the host cell surface receptor angiotensin-converting enzyme 2 (ACE2). The spike protein is a critical target for neutralizing antibodies and relevant vaccines. SARS-CoV-2 variants of concern (VOC) mainly evolve through mutations in the spike gene, particularly in the receptor-binding domain (RBD) of the spike protein, under immune selection pressure induced by virus transmission or vaccination in humans [3]. For instance, the Omicron variants have more than 35 mutations in the spike and 15 mutations in the RBD compared to the ancestral strain, significantly enhancing its transmission and rendering it resistant to neutralizing antibodies (NAbs) induced by infection or vaccination [4]. Unfortunately, continued mutation of the SARS-CoV-2 Omicron variant spike has resulted in the emergence of several new subvariants that have increased resistance to neutralization by sera from patients who have received mRNA vaccination, have been infected with BA.1, or have been infected with BA.4/5 [4].

In addition to the spike protein, mutations in accessory proteins are also highly frequent in most VOC, which may impact their secondary structure and biological function [2, 5]. These accessory proteins play crucial roles in host immune evasion and virus pathogenesis [6, 7]. Open reading frames (ORF) 7a, ORF7b and ORF8 can inhibit IFN-I signaling, which is the host's first line of defense against invading viruses [6]. Additionally, ORF8 has been proven to interact with major histocompatibility complex I, impairing the activity of antigen-presenting cells [6]. ORF7a and ORF7b were recently reported to interact with CD14^+^ monocytes, leading to a decrease in their antigen-presenting ability and triggering a significant upregulation of pro-inflammatory cytokines such as IL-6, IL-1β, IL-8, and TNF-α [8, 9]. During the early COVID-19 epidemic, SARS-CoV-2 AS with a large 382-nucleotide deletion (Δ382), leading to the truncation of ORF7b and removal of ORF8 transcription, was reported to have higher replicative fitness in vitro and similar viral load compared to the wild type (WT) [10]. Clinical analysis revealed that COVID-19 patients infected by Δ382 AS had higher concentrations of IFN-γ and lower concentrations of the chemokines IP-10 (CXCL10), MCP-1 (CCL2), and MIP-1β (CCL4), and lower odds of developing hypoxia compared to patients infected by the WT virus [11], indicating the potential pathogenesis of ORF8.

An 872 bp nucleotide deletion encompassing ORF7a, ORF7b and ORF8 has been reported in SARS-CoV-2 Delta variant AY.4 isolates [12]. Interestingly, Delta variant AY.4 isolates without the ORF7a, ORF7b, and ORF8 genes exhibit similar transmissibility to the parental AY.4 lineage [12]. A recent study created SARS-CoV-2 AS with the simultaneous deletion of ORF7a, ORF7b, and ORF8 (∆678 AS) [13]. Experimental data showed that the ∆678 AS developed plaques similar to the WT-AS but had reduced lung viral loads after intranasal infection of BALB/c mice relative to WT-AS [13]. However, the effects of the simultaneous deletion of ORF7a, ORF7b, and ORF8 on pathogenesis and the host immune response in humans are unknown. Interestingly, an 871 bp nucleotide deletion that encompasses ORF7a, ORF7b, and ORF8 has also been observed in Omicron variants BA.2, which caused the SARS-CoV-2 outbreak in Maoming city, China. Herein, the clinical characteristics and immune response of patients with Omicron variants BA.2 with or without the 871 bp nucleotide deletion were comparatively analyzed to explore the effect of simultaneous deletion of ORF7a, ORF7b, and ORF8 on pathogenesis and the host immune response.

## Method and materials

### Study cohorts

This study was conducted in compliance with the Declaration of Helsinki and was approved by the Maoming People's Hospital (Approval No. 2021-hs-43) and Dongguan Ninth People's Hospital (Approval No. 2022-8). COVID-19 was confirmed based on positive results of reverse transcription-polymerase chain reaction (RT-PCR) of nasopharyngeal samples. Whole blood was collected from enrolled COVID-19 patients after obtaining informed consent. Clinical parameters, including sex, age, vaccination history and whole blood counts, were obtained from medical records.

### Complete genome sequencing and analysis

Total ribonucleic acid (RNA) was isolated from nasopharyngeal swab specimens using a DNA/RNA extraction kit (Tianlong, China). The RNA was then subjected to whole-genome amplification using the ultrasensitive complete genome capture kit (Beijing MicroFuture Technology Company, China). A sequencing library was constructed using the Nextera XT DNA library preparation kit (Illumina, USA) and sequenced using the 100-cycle version MiniSeq Reagent Kit (Illumina, USA) on the Next Generation MiniSeq sequencer (Illumina, USA). Raw NGS reads were trimmed using Trimmomatic v0.39 to remove adaptors and low-quality bases. Genome sequences were then assembled, and consensus sequences were obtained using the BWA-MEM algorithm in UGENE v.33.

### Phylogenetic analyses

The complete genomes of representative SARS-CoV-2 variants were randomly downloaded from the GISAID database. Then, genome sequence alignment was performed using MAFFT, and IQ-TREE was used to generate the maximum likelihood tree.

### RT-PCR and Sanger sequencing

Specific primers flanking the deleted region were designed using primer designer software. The nucleic acid was amplified using the One-step RT-PCR System (Invitrogen, USA), and the amplified products were visualized by capillary electrophoresis to confirm the presence of amplified bands. The products were then sent for Sanger sequencing, and the results were edited and assembled using the DNAStar and MEGA X software.

### Cytokine detection

Plasma cytokine detection was performed using a commercial Multi-cytokine detection kit (Cellgene Biotech, China). The cytokines detected included interleukin (IL)-2, IL-4, IL-6, IL-10, tumor necrosis factor (TNF)-α, interferon (IFN)-γ, IL-17A, IL1-β, IL-5, IL-12P70, IFN-α, and IL-8, according to the manufacturer's instructions. Briefly, 20 μL of capture microsphere solution, 25 μL of tested plasma sample or diluted standard sample, and 25 μL of fluorescence detection solution were added into a tube in turn and incubated for 2.5 h. Then, 1 mL of phosphate buffer solution (PBS) was added, followed by centrifugation at 200 g for 5 min. The supernatant was then discarded, and the precipitant was resuspended using 100 mL of PBS. The cytokine concentration in the solution was detected using a BD FACSCalibur™ flow cytometer (BD Biosciences, USA), and the results were subsequently loaded onto the FCAP Array™ version 3.0 (BD Biosciences, USA) software to determine the cytokine concentrations based on a logistic curve-fitting equation, which was generated using the serial diluted standard sample.

### Binding antibody IgG titers detection

SARS-CoV-2 ancestor, Omicron variants BA.2 and BA.5 receptor binding domain (RBD) specific antibody IgG titers were measured using enzyme-linked immunosorbent assay (ELISA), as previously described [1, 14]. Briefly, the ELISA assay for RBD-specific IgG antibodies was performed as follows: first, 100 μL of the appropriate concentration of RBD was added to each well of the ELISA plate and incubated at 4 °C overnight. The plate was then washed three times with phosphate buffer solution containing 0.1% tween 20 (PBST) before adding threefold serially diluted plasma (starting dilution from 1:20) to each well, followed by incubation at 37 °C for 1 h. After washing again, 100 μL of diluted anti-human IgG antibody conjugated with HRP (1:10,000, Southern Biotech, USA) was added to each well and incubated for 1 h. Then, 50 μL of TMB substrate (Neobioscience, China) was added to each well, and the plates were incubated for 10–15 min in the dark. Finally, the OD value was measured at 450 nm using a microplate absorbance reader (Tecan Sunrise, Switzerland). The antibody endpoint titer was determined based on the highest dilution that gave an OD value higher than the mean plus three standard deviations (SD) of the OD values of 3 serum pools from 45 stored serums at the same dilution collected from healthy individuals in 2019.

### Pseudovirus neutralization assays

Pseudovirus neutralization assays were performed as previously described [15]. Briefly, the lentivirus-based pseudotyped SARS-CoV-2 (including ancestor, Omicron BA.2, and BA.5) was produced in HEK293T cells by co-transfection with lentivirus pseudotyped system backbone plasmids and a codon-optimized spike protein-expressing plasmid. Pseudovirus-containing supernatants were collected 60 h after transfection and titrated by infecting 293 T-ACE2 cells. Serum samples were serially diluted three-fold, starting at a 1:10 dilution, and then co-incubated with 800 50% tissue culture half infective dose pseudovirus supernatants in 96-well microplates (JETBIOFIL, China) for 1 h at 37 °C. Subsequently, 100 μL of the mixture from each well was transferred to white 96-well cell culture plates (Corning, USA), and 20,000 293 T-hACE2 cells per well were added in sequence. After incubating for 72 h at 37 °C and 5% CO_2_, the reduction in relative luminescence units compared to virus-infected untreated control cells was determined using the Bio-LiteTM Luciferase Assay (Vazyme, China) on a Cytation 1 cell imaging multi-mode reader (Biotek, USA). The 50% inhibitory dilution (ID50) values for neutralization were calculated by fitting sigmoidal dose–response curves to the respective data using GraphPad Prism v.9.2.

### Effector T cells frequencies detection

SARS-CoV-2 ancestral strain and Omicron spike, as well as ancestral strain NP-specific effector T cells, were detected using an interferon-γ (IFNγ) enzyme-linked immunospot (ELISPOT) assay as previously described [16]. Briefly, 2 × 10^5^ fresh PBMCs were added to each well of an anti-IFNγ pre-coated ELISPOT plate (Dakewe Biotech, China) and co-cultured with 50 ng overlapping peptide pools of SARS-CoV-2 ancestral strain or Omicron variant spike, or ancestral strain NP (Genscript, China) for 24 h, using dimethyl sulfoxide (Sigma, USA) as a negative control (NC). For the positive control, 2 × 10^4^ PBMCs were stimulated with staphylococcal enterotoxin B (1 µg/mL, Merck, Germany). After completing the chromogenic reaction, spots were counted using the ImmunoSpot® S6 UV Analyzer (Cellular Technology Limited, USA). The spot-forming units (SFU) of each well were determined by subtracting the spots of the unstimulated wells from the peptide-stimulated wells. The SFU of each sample was expressed as SFU/10^6 PBMCs.

### Detection of SARS-CoV-2 specific memory B cells (MBCs)

MBCs were determined using ELISPOT assay, as previously described [17]. Briefly, 1.5 × 10^6^ PBMCs were stimulated with 1 μg/mL R848 (InvivoGen, USA) and 100 IU/mL recombinant human IL-2 (Peprotech, USA) in RPMI-1640 (Gibco, USA) supplemented with 10% fetal bovine serum (Guangzhou Cellcook Biotech, China) for three days. ELISPOT plates (Mabtech, USA) were pre-coated with 10 μg/mL of SARS-CoV-2 S1 (Dongkang Biotech, China), RBD (Dongkang Biotech, China), and Omicron-RBD (Fapon Biotech, China), along with 10 μg/mL anti-human IgG (Jackson ImmunoResearch, USA). Next, 100,000 activated cells were added to the ELISPOT plates and incubated at 37 °C in 5% CO_2_ for 18 h. To exclude nonspecific binding, control wells without coating target protein and anti-human IgG were also incubated with 100,000 pre-stimulated cells. After washing the plates six times with PBST, 100 μL of HRP-labeled Goat Anti-Human IgG (Beyotime, China) in PBS supplemented with 5% FBS was added to the plate wells and incubated for 2 h at room temperature. Spots were then developed with a 3-amino-9-ethylcarbazole (AEC) substrate (BD Biosciences, USA) according to the manufacturer's instructions. Finally, spots were counted using the ImmunoSpot S6 UV Analyzer (Cellular Technology Limited, USA). The SFU of each well was determined by subtracting spots of the same sample in the control wells. The SFU of each sample was calculated using the means of duplicate wells and expressed as SFU/10^6^ PBMCs.

### Statistical analysis

All statistical analyses were performed using GraphPad Prism (version 9.2). Binding antibody and neutralizing antibody titers were expressed as geometric mean titers (GMTs) with a 95% confidence interval (CI). Continuous variables are presented as median (interquartile range [IQR]), while categorical variables are described as counts and percentages. Differences in proportions between two groups were determined using the Pearson Chi-square test. Independent group Mann–Whitney U tests were employed to compare continuous variables between groups. A two-sided *p*-value less than 0.05 was considered statistically significant.

## Results

### SARS-CoV-2 sequence information

**Fig. 1:**
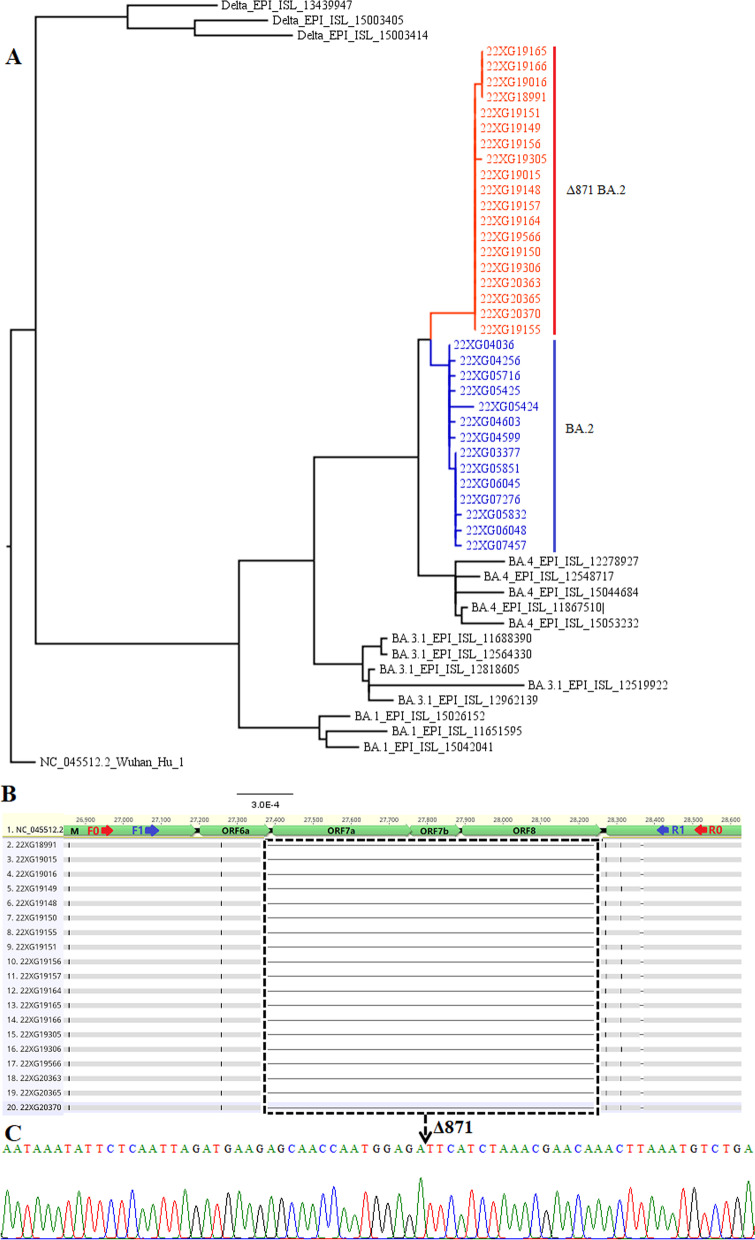
Complete genome characteristics of the SARS-CoV-2 Omicron subvariants. **A** The maximum likelihood phylogeny based on the full-length genome sequences of SARS-CoV-2 Omicron subvariants. **B** Alignment of the consensus sequences of isolates with 871 nt deletions to the SARS-CoV-2 reference genomic sequence NC_045512.2 (n = 20), the red and blue arrow mark two pairs of Sanger sequencing primers, respectively. **C** Sanger sequencing results flanking the 871-nt deletion of the Omicron subvariants Δ871 BA.2, the dotted arrow indicates the 871-nt deletion

An outbreak of COVID-19 occurred in Maoming City between July 8 and July 14, 2022, and subsequent analysis confirmed that it was caused by Omicron variants BA (Fig. 1A). Alignment analysis of the whole genome from 19 patients showed large sequences (800–900 bp) deletion spanning ORF7a, ORF7b and ORF8 genes (Fig. 1B). To confirm these unexpected results, two pairs of specific primers flanking the deleted region were used to amplify the genome sequences, which were then subjected to Sanger sequencing (Fig. 1B, Additional file 1: Table 1). The sequencing results showed a clear linkage between nucleotide positions 27,380 and 28,250 of the reference genome, indicating a deletion of 871 bp nucleotides in Omicron variants BA.2 (∆871 BA.2) might have been responsible for the deletion of ORF7a, ORF7b and ORF8 (Fig. 1C).

**Table 1:** Baseline characteristics of enrolled Omicron ∆872 BA.2 breakthrough infected patients

Variables (n [%] or median [IQR])	Juveniles (n = 21)	Adults (n = 7)	*p*-value
Median Age (Range)	16(8–17)	43(36–56)	0.003
Sex			
Female	19(90.48%)	5(71.43%)	0.213
Male	2(9.52%)	2(28.57%)	
Vaccination status			
Two doses of InV	21(100%)	2(28.6%)	< 0.0001
Three doses of InV	0	5(71.4%)	
Hospital stay days	9(8–9)	7(6–9)	0.126
CT value(NP)	19.53(18.17–23.87)	19.43(18.66–23.20)	0.756
CT value(ORF1ab)	19.87(17.04–25.81)	19.52(17.46–24.28)	0.717
Symptoms			
Fever	21(100%)	5(71.43%)	0.568
Sore throat	5(23.81%)	2(28.57%)	
Cough	5(23.81%)	4(57.14%)	
Fatigue	9(42.86%)	1(14.29%)	
Headaches	9(42.86%)	1(14.29%)	
Diarrhea	1(4.76%)	0	
Stuffy nose	5(23.81%)	1(14.29%)	
Myalgia	1(4.76%)	1(14.29%)	
Nausea and vomiting	2(9.52%)	0	
Severity			
Asymptomatic	0	1(14.29%)	0.078
Mild	21(100%)	6(85.71%)	
Underlying diseases			
Diabetes	1(4.76%)	0	0.157
Hypertension	0	1(14.29%)	
Complete blood counts			
WBC(10^9^/L)	7.30(5.23–8.61)	6.23(5.94–7.83)	0.788
RBC(10^12^/L)	4.49(4.055–4.88)	4.49(4.29–4.90)	0.547
HGB(g/L)	126.0(110.5–137.0)	134.0(129.0–157.0)	0.045
Platelet(10^9^/L)	265.0(183.0–294.0)	203.0(193.0–232.0)	0.336
Neutrophils(10^9^/L)	4.76(2.80–7.12)	4.69(4.29–5.54)	0.905
Monocytes(10^9^/L)	0.5(0.46–0.64)	0.49(0.41–0.71)	0.825
Lymphocyte(10^9^/L)	0.9(0.61–1.45)	0.93(0.52–1.51)	0.926
Eosinophils(10^9^/L)	0.13(0.08–0.28)	0.12(0.07–0.18)	0.460
Basophils(10^9^/L)	0.01(0.01–0.02)	0.01(0–0.01)	0.430

*IQR* interquartile rang; *InV* inactivated vaccine; *WBC* white blood cell; *RBC*,Red blood cells; *HGB* Hemoglobin

### Patient information and clinical characteristics

During the outbreak, a total of 28 individuals were confirmed to have COVID-19 based on positive SARS-CoV-2 RNA results from nasopharyngeal samples. Table 1 lists the detailed information on the confirmed patients. Of them, 4 were male and 24 were female. Among them, 21 were adolescents, with a median age of 16, while 7 were adults, with a median age of 43. All adolescent patients had completed a two-dose inactivated vaccine scheme and were infected by ∆871 BA.2 at a median of 11 months post-vaccination. Two adult patients had completed a two-dose inactivated vaccine scheme, and 5 had completed a homologous third inactivated vaccine booster before infection with ∆871 BA.2 (Table 1). All adult patients were infected 5–6 months after their latest vaccination. Further analysis between ∆871 BA.2 infected adolescents and adults showed that blood hemoglobin levels were higher in adult patients than in juvenile patients (Table 1). In addition, there were no significant differences in other clinical parameters, including hospital stay days, peak virus load, symptoms, clinical severity and underlying diseases between ∆871 BA.2 infected adolescents and adults (Table 1).

For comparative purposes, we also included 40 COVID-19 patients infected with the wild-type BA.2 strain in Dongguan from February 25, 2022, to March 21, 2022, who did not show any deletion in ORF7a, ORF7b, and ORF8 genes (Fig. 1A) in this study [1]. There were no significant differences in age, sex, vaccination history, underlying diseases, or symptoms between the WT BA.2 infected patients and the ∆871 BA.2 infected adult patients (Table 2). All patients were infected with the WT BA.2 variant at a median of 6 months after their latest vaccination. Comparative analysis showed that the peak viral load in nasopharyngeal samples was similar between the ∆871 BA.2 infected adult patients and the WT BA.2 infected adult patients (Table 2). Interestingly, the hospitalized days for ∆871 BA.2 infected patients were shorter than that for WT BA.2 infected patients (Table 2). Additionally, the asymptomatic infection rate in enrolled WT BA.2 infected patients was higher than in the ∆871 BA.2 infected patients (Table 2). Further, ∆871 BA.2 infected patients exhibited lower platelet counts and higher eosinophil counts than BA.2 infected patients (Table 2), and no significant differences in other clinical parameters were observed between the two groups in this study (Table 2).

**Table 2:** Baseline characteristics of enrolled Omicron variants ∆872 BA.2 and WT BA.2 breakthrough infected adult patients

Variables (n [%] or median [IQR])	BA.2.2 Infected Adult Patients (n = 40)	BA.2.3 Infected Adult Patients (n = 7)	*p*-value
Age (years)	35(31–45)	43(36–56)	0.381
Sex			
Female	15(37.5%)	5(71.4%)	0.094
Male	25(62.5%)	2(28.6%)	
Vaccination status			
Two doses of InV	19(47.5%)	2(28.6%)	0.353
Three doses of InV	21(52.5%)	5(71.4%)	
Hospital stay days	13(9.75–15)	7(6–9)	0.020
CT value(NP)	17.0(15.0–20.0)	19.43(18.66–23.20)	0.070
CT value(ORF1ab)	17.0(14.0–20.89)	19.52(17.46–24.28)	0.256
Symptoms			
Fever	32(80%)	5(71.4%)	0.898
Sore throat	13(32.5%)	2(28.6%)	
Cough	21(52.5%)	4(57.1%)	
Fatigue	13(32.5%)	1(14.3%)	
Headaches	12(30%)	1(14.3%)	
Diarrhea	8(20%)	0	
Stuffy nose	7(17.5%)	1(14.3%)	
Myalgia	2(5%)	1(14.3%)	
Nausea and vomiting	1(2.5%)	0	
Severity			
Asymptomatic	18(45%)	1(14.3%)	0.047
Mild	22(55%)	6(85.7%)	
Underlying diseases			
Cardiovascular diseases	3(7.5%)	0	0.405
Hypertension	1(2.5%)	1(14.29%)	
Pulmonary inflammation	1(2.5%)	0	
Pulmonary bulla	1(2.5%)	0	
Complete blood counts			
WBC(10^9^/L)	5.81(4.41–8.45)	6.23(5.94–7.83)	0.512
RBC(10^12^/L)	4.89(4.60–5.29)	4.49(4.29–4.90)	0.086
HGB(g/L)	140.0(125.8–149.0)	134.0(129.0–157.0)	0.725
Platelet(10^9^/L)	260.0(225.5–306.3)	203.0(193.0–232.0)	0.041
Neutrophils(10^9^/L)	4.05(3.06–6.56)	4.69(4.29–5.54)	0.827
Monocytes(10^9^/L)	0.48(0.335–0.58)	0.49(0.41–0.71)	0.386
Lymphocyte(10^9^/L)	1.06(0.75–1.48)	0.93(0.52–1.51)	0.582
Eosinophils(10^9^/L)	0.04(0.01–0.09)	0.12(0.07–0.18)	0.027
Basophils(10^9^/L)	0.01(0.01–0.01)	0.01(0–0.01)	0.280

*IQR* interquartile rang; *InV* inactivated vaccine; *WBC* white blood cell; *RBC* Red blood cells; *HGB* Hemoglobin

### Inflammatory response in ∆871 BA.2 and BA.2 infected patients

Several studies have reported that ORF7a, ORF7b and ORF8 can inhibit IFN-I signaling and promote inflammatory responses [6, 9]. To explore the effects of the deletion of ORF7a, ORF7b, and ORF8 on the inflammatory response, inflammatory cytokines were detected at the acute phase of COVID-19 patients infected by ∆871 BA.2 or WT BA.2. The results showed that plasma levels of IL-10, IL-17A, and IL-8 were higher in ∆871 BA.2 infected adult patients as compared to BA.2 infected adult patients (Table 3). However, the plasma concentration of IFN-γ was lower in ∆871 BA.2 infected adult patients than in BA.2 infected adult patients (Table 3). To explore the effects of age, comparative analysis of ∆871 BA.2 infected adolescents and adults was performed, and the results showed ∆871 BA.2 infected adolescents had higher CRP and lower IL-8 than adult patients (Table 4).

**Table 3:** Comparative analysis of inflammatory cytokines between Omicron variants ∆872 BA.2 and WT BA.2 breakthrough infected adult patients

Variables (median [IQR])	Normal reference range	BA.2.2 Infected adult Patients (n = 40)	∆872 BA.2.3 Infected adult Patients (n = 7)	*p*-value
CRP (μg/mL)	0–8.2	1.815(0.43–3.81)	2.95(1.26–4.51)	0.309
IL-2 (pg/mL)	≤ 11.4	0.74(0.49–1.23)	1.23(0.79–2.06)	0.057
IL-4 (pg/mL)	≤ 12.9	1.93(1.57–2.42)	2.39(1.49–3.01)	0.212
IL-6 (pg/mL)	≤ 20.0	3.83(3.13–7.02)	4.72(4.08–8.56)	0.135
IL-10 (pg/mL)	≤ 5.9	1.9(1.45–2.45)	3.18(1.94–3.89)	0.021
TNF-α (pg/mL)	≤ 5.5	1.83(1.38–2.15)	2.07(1.83–2.75)	0.054
IFN-γ(pg/mL)	≤ 17.3	4.31(2.28–6.18)	2.22(1.73–2.86)	0.035
IL17A (pg/mL)	≤ 20.6	2.07(1.71–2.78)	3.52(2.0–4.31)	0.031
IL1-β (pg/mL)	≤ 12.1	1.09(0.77–2.05)	1.52(1.15–1.91)	0.306
IL-5 (pg/mL)	≤ 3.4	0.68(0.48–0.89)	0.73(0.49–0.97)	0.614
IL12P70 (pg/mL)	≤ 3.2	1.63(1.40–2.17)	1.86(1.11–2.01)	0.710
IFN-α (pg/mL)	≤ 7.9	2.20(1.22–5.725)	3.67(1.62–18.11)	0.169
IL-8 (pg/mL)	≤ 21.4	3.92(2.73–12.49)	10.26(7.18–29.53)	0.039

**Table 4:** Comparative analysis of inflammatory cytokines between Omicron variants ∆872 BA.2 breakthrough infected adolescents and adults

Variables (median [IQR])	Normal reference range	Adolescents (n = 21)	Adults (n = 7)	*p*-value
CRP	0–8.2	8.02(5.02–12.92)	2.95(1.26–4.51)	0.019
IL-2	≤ 11.4	0.9(0.69–1.52)	1.23(0.79–2.06)	0.473
IL-4	≤ 12.9	2.16(1.65–2.63)	2.39(1.49–3.01)	0.668
IL-6	≤ 20.0	3.83(3.3–4.59)	4.72(4.08–8.56)	0.056
IL-10	≤ 5.9	2.36(1.86–3.38)	3.18(1.94–3.89)	0.368
TNF-α	≤ 5.5	1.99(1.6–2.32)	2.07(1.83–2.75)	0.370
IFN-γ	≤ 17.3	2.32(1.82–2.64)	2.22(1.73–2.86)	0.896
IL-17A	≤ 20.6	5.54(3.33–2.48)	3.52(2.0–4.31)	0.104
IL1-β	≤ 12.1	1.27(0.91–2.26)	1.52(1.15–1.91)	0.907
IL-5	≤ 3.4	0.59(0.45–0.9)	0.73(0.49–0.97)	0.275
IL-12P70	≤ 3.2	1.86(1.4–2.33)	1.86(1.11–2.01)	0.440
IFN-α	≤ 7.9	4.74(2.47–11.48)	3.67(1.62–18.11)	0.928
IL-8	≤ 21.4	2.87(1.33–7.66)	10.26(7.18–29.53)	0.002

### SARS-CoV-2 specific antibody in ∆871 BA.2 or WT BA.2 infected patients

**Fig. 2:**
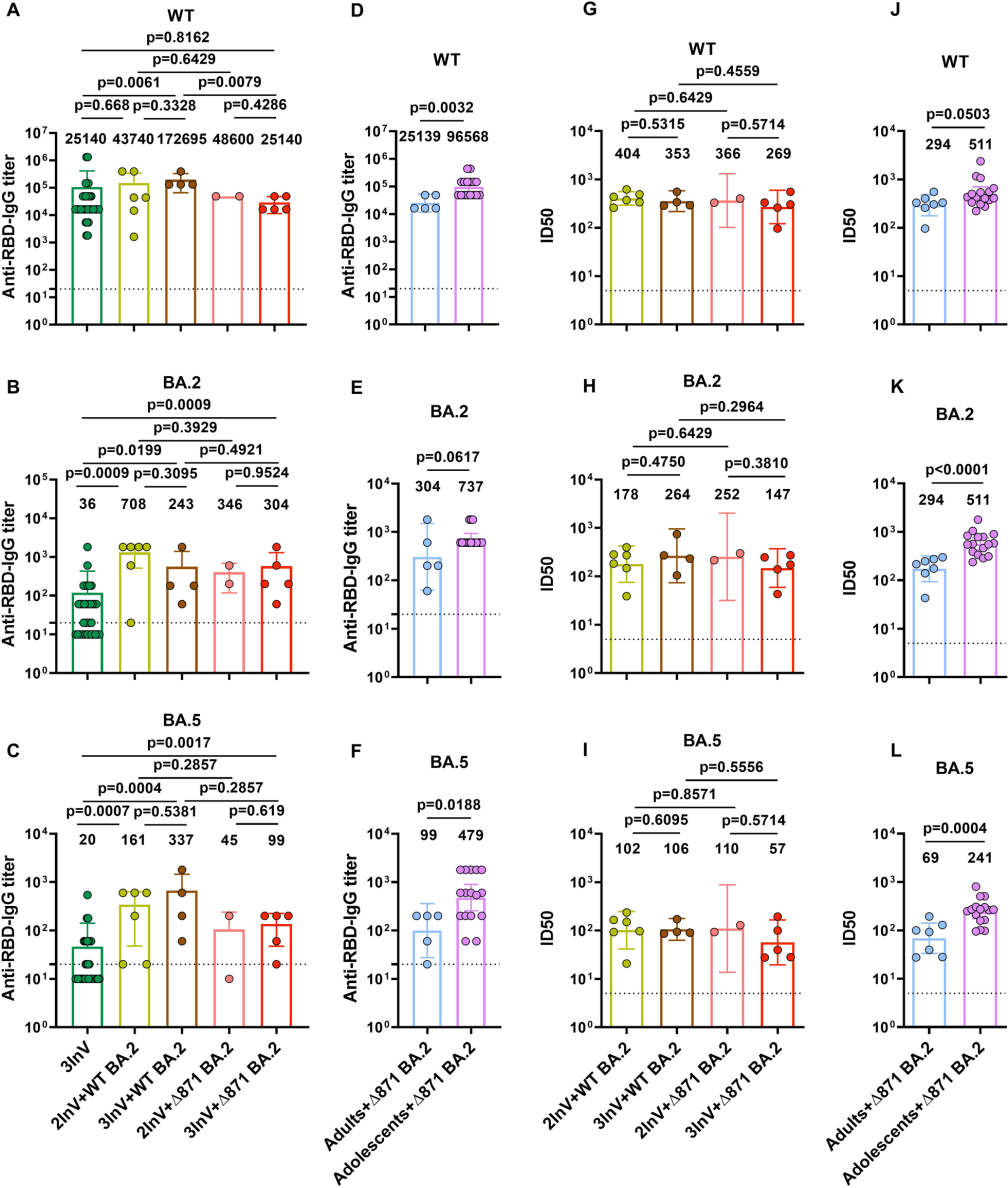
The antibody titers against AS, Omicron BA.2 and BA.5 in the third homologous inactivated vaccine boosted healthy individuals, ∆871 BA.2 and BA.2 breakthrough infected patients. **A**–**C** Binding antibody IgG titers against AS RBD (**A**), Omicron BA.2 RBD (**B**) and BA.5 RBD (**C**) in third homologous inactivated vaccine boosted healthy individuals, ∆871 BA.2 and BA.2 breakthrough infected patients with completion of two doses inactivated vaccine scheme or homologous booster. **D**–**F** Binding antibody IgG titers against AS RBD (**D**), Omicron BA.2 RBD (**E**) and BA.5 RBD (**F**) in ∆871 BA.2 and BA.2 breakthrough infected adolescents and adults. **G**–**I** neutralizing antibodies (NAbs) titers against AS (**G**), Omicron BA.2 (**H**) and BA.5 (**I**) in ∆871 BA.2 and BA.2 breakthrough infected patients with completion of two doses inactivated vaccine scheme or homologous booster. **J**–**L** NAbs titers against AS (**J**), Omicron BA.2 (**K**) and BA.5 (**L**) in ∆871 BA.2 and BA.2 breakthrough infected adolescents and adults. Data presented were from 3-independent experiments and analyzed using the two-sided Mann–Whitney U-test. Error bars represent geometric mean titers (GMTs) and 95% confidence interval (CI)

Here, the effects of ORF7a, ORF7b, and ORF8 simultaneous deletion on the humoral immunity were further dissected. Firstly, plasma binding antibodies IgG titers against AS, BA.2 and BA.5 RBD were detected in ∆871 BA.2 or WT BA.2 breakthrough infected patients. The results demonstrated comparable binding antibody IgG titers against AS RBD in ∆871 BA.2 and WT BA.2 infected adult patients relative to homologous inactivated vaccine-boosted healthy individuals (Fig. 2A). However, the binding antibodies IgG titers against BA.2 and BA.5 RBD were higher than that of homologous inactivated vaccine-boosted healthy individuals (Fig. 2B, C). Moreover, ∆871 BA.2 and WT BA.2 infected adult patients had comparable binding antibodies IgG titers against AS, BA.2 and BA.5 RBD (Fig. 2A–C), and the binding antibodies IgG directed to AS, BA.2 and BA.5 RBD were higher in ∆871 BA.2 breakthrough infected adolescent patients compared to adult patients (Fig. 2D–F). Accordingly, the neutralizing antibodies titers against AS, BA.2 and BA.5 in ∆871 BA.2 infected adult patients were similar to that in WT BA.2 infected adult patients (Fig. 2G–I). In addition, the neutralizing antibodies titers AS, BA.2 and BA.5 in ∆871 BA.2 infected adult patients were lower than in ∆871 BA.2 infected juvenile patients (Fig. 2J–L).

### Effector T cells immunity response in ∆871 BA.2 and WT BA.2 infected patients

**Fig. 3:**
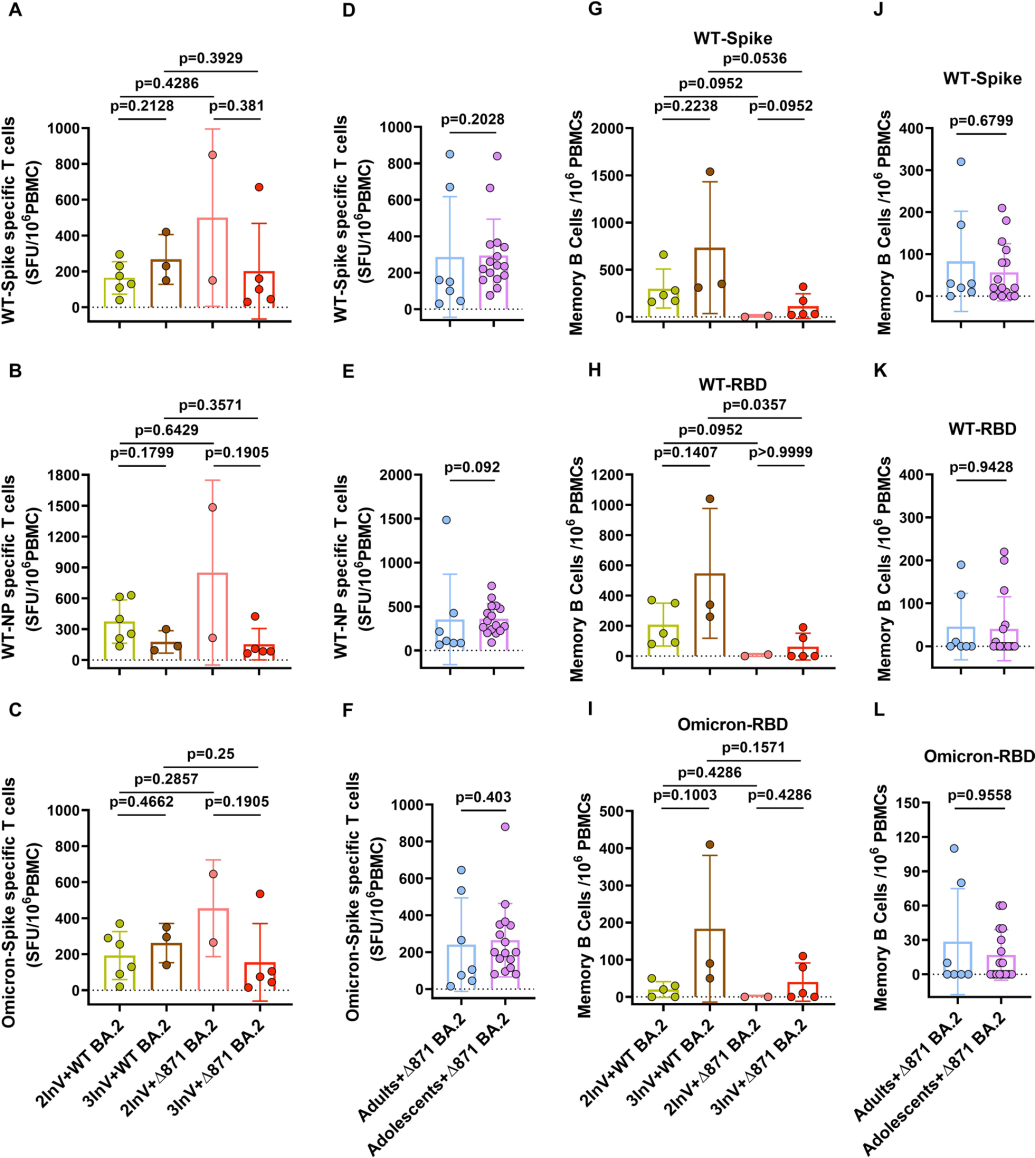
Effector T cells and memory B cells frequency in ∆871 BA.2 and BA.2 breakthrough infected patients. **A**–**C** Effector T cells frequency against AS spike (**A**), AS NP (**B**), and Omicron spike (**C**) in ∆871 BA.2 and BA.2 breakthrough infected patients with completion of two doses inactivated vaccine scheme or homologous booster. **D**–**F** Effector T cells frequency against AS spike (**D**), AS NP (**E**) and Omicron spike (**F**) in ∆871 BA.2 and BA.2 breakthrough infected adolescents and adults. **G**–**I** Memory B cell frequency against AS spike (**G**), AS receptor binding domain (RBD) (**H**) and Omicron RBD (**I**) in ∆871 BA.2 and BA.2 breakthrough infected patients with completion of two doses inactivated vaccine scheme or homologous booster. **J**–**L** Memory B cell frequency against AS spike (**J**), AS RBD (**K**) and Omicron RBD (**L**) in ∆871 BA.2 and BA.2 breakthrough infected adolescents and adults. Error bars represent the median (interquartile range [IQR]). Data presented were from 3-independent experiments and analyzed using the two-sided Mann–Whitney U-test

Considering that effector T cells play a crucial role in the clearance of virus-infected cells and are a principal arm of the immune response in the recovery of COVID-19 patients [18], we evaluated effector T cells frequency against AS spike and nucleocapsid protein (NP), Omicron spike. The results showed no significant difference in the frequency of effector T cells against AS spike and nucleocapsid protein (NP) or Omicron spike between ∆871 BA.2 and WT BA.2 breakthrough infected patients who had completed two doses of the inactivated vaccine or received a third homologous booster (Fig. 3A–C). Additionally, there was no significant difference in effector T cell frequency against AS and Omicron spike between ∆871 BA.2 breakthrough infected adolescents and adults (Fig. 3D–F).

### MBCs in ∆871 BA.2 and WT BA.2 infected patients

MBCs constitute another essential component of durable protective immunity, capable of rapidly proliferating and evolving to generate antibodies against SARS-CoV-2 or its variants upon re-exposure [19]. Researchers evaluated SARS-CoV-2-specific MBCs using the ELISPOT assay after stimulating them with agonist R848 and Interleukin (IL)-2 [20]. The results revealed no significant differences in MBC frequency against the AS spike and RBD or Omicron RBD in individuals who had received either two doses of the inactivated vaccine or a third homologous booster, whether they had the ∆871 BA.2 or WT BA.2 BIPs (Fig. 3G–I). Furthermore, MBCs targeting the AS spike and RBD, as well as the Omicron RBD, were found to be comparable between ∆871 BA.2 breakthrough-infected adolescents and adults (Fig. 3J–L).

## Discussion

Multiple studies have shown that ORF7a, ORF7b, and ORF8 play a role in the pathogenesis of SARS-CoV-2, and their deletion can impact virulence [13]. Consistent with a previous study [10], a local COVID-19 outbreak caused by Omicron variant BA.2 with the deletion of ORF7a, ORF7b and ORF8 suggests that ∆871 BA.2 retains the ability to efficiently infect and transmit between humans. Our data further demonstrated that the hospitalization days of ∆871 BA.2 BIPs were shorter than WT BA.2 BIPs, indicating a rapid clearance of BA.2 after deletion of accessory proteins ORF7a, ORF7b and ORF8. The clinical symptoms were comparable between ∆871 BA.2 and WT BA.2 BIPs. Although some cytokines in the acute stage differed between ∆871 BA.2 and WT BA.2 BIPs, the levels of all detected cytokines were within the normal reference range. Collectively, these data suggest that the combined deletion of ORF7a, ORF7b and ORF8 might have little impact on the clinical symptoms of BA.2 BIPs. Importantly, breakthrough infection with ∆871 BA.2 may not significantly influence the host humoral and cellular immunity against SARS-CoV-2 variants, as compared to WT BA.2.

The speed of viral clearance is dependent on the intrinsic characteristics of SARS-CoV-2 variants and baseline immunity against SARS-CoV-2 [21]. Protective immunity prior to infection has been shown to significantly benefit rapid viral clearance in Delta variants [22]. Compared to Delta variants, Omicron variants have lower virulence [23]. A study on Omicron subvariants BA.1 demonstrated that the duration of viral shedding of Omicron variants was not significantly affected by baseline-specific immunity [24]. Here, our study found that the combined deletion of ORF7a, ORF7b, and ORF8 might change the intrinsic characteristics of Omicron subvariants BA.2, contributing to more rapid viral clearance. In addition, the deletion of ORF7a, ORF7b, and ORF8 in AS reduces viral loads in the lungs after intranasal infection of BALB/c mice [13]. Moreover, a 382-nucleotide deletion in AS results in the expression of a novel ORF7b-ORF8 fusion protein and the elimination of ORF8, leading to a mild infection [10, 11].

Contrary to a previous study that demonstrated the deletion of ORF8 in AS resulted in higher concentrations of IFN-γ and lower concentrations of chemokines IP-10 (CXCL10), MCP-1 (CCL2), and MIP-1β (CCL4) [11], our research discovered that plasma IL-10, IL-17A, and IL-8 levels were higher in ∆871 BA.2 infected adult patients compared to BA.2 infected adult patients. Multiple studies have shown that IL-10, IL-17A, and IL-8 levels positively correlate with clinical severity in SARS-CoV-2-infected patients [25]. Therefore, we hypothesize that the observed difference may be related to the higher rate of asymptomatic infection in WT BA.2 breakthrough infected patients compared to ∆871 BA.2 breakthrough infected patients (Table 2). However, plasma IFN-γ concentrations were lower in ∆871 BA.2 infected adult patients than in BA.2 infected adult patients. Notably, these statistically different cytokine levels remained within normal limits, which may signify a significant reduction in pathogenesis and pro-inflammatory effects of Omicron variants [23]. Furthermore, all enrolled patients had completed the full vaccination scheme, which likely provided additional protective immunity, promoting virus clearance and leading to a lower inflammatory response.

The emergence of new Omicron subvariants highlights the need for variant-specific vaccines to improve immunity against the currently dominant variant. Several novel Omicron subvariants, such as BA.4/5, BA.2.75, BF.7, BQ.1, and BQ.1.1, have emerged from the BA.2 lineage [4]. Therefore, an Omicron BA.2-based vaccine could be a better candidate for enhancing immunity against the current dominant Omicron subvariants. This has been demonstrated in both naïve and vaccinated individuals [26]. Here, our study further showed that ∆871 BA.2 breakthrough infected patients had comparable antibody IgG, neutralizing antibodies titers, MBCs, and effector T cells to WT BA.2 breakthrough infected patients, indicating that ∆871 BA.2 infection after full vaccination could induce comparable protective humoral and cellular immunity against both BA.2 and BA.5, as with WT BA.2 [26].

Several studies have shown that Omicron variants have lower virulence than AS and Delta variants due to reduced cell–cell fusogenicity and impaired replication in lower airway epithelial cells [4]. The deletion of ORF7a, ORF7b, and ORF8 further decreases the pathogenicity of BA.2, making it a potential candidate for a live attenuated vaccine. Live attenuated vaccines can mimic natural infections, inducing longer-lasting protection and providing broader immunity effective against current and future variants compared to existing licensed mRNA, viral vector, subunit protein, and inactivated vaccines [1, 27, 28]. Therefore, a live attenuated vaccine based on BA.2 could be a valuable addition to the current vaccine arsenal.

## Conclusions

In conclusion, the deletion of ORF7a, ORF7b, and ORF8 may reduce the duration of hospitalization for Omicron variant BA.2 BIPs. However, these deletions do not appear to affect clinical severity or the protective humoral and cellular immunity of BIPs, indicating that this modified BA.2 could be a potential candidate for an Omicron variant-specific live attenuated vaccine.

## Supplementary Information

**Additional file 1**. Primer pairs used for amplification and Sanger sequencing of detected region.

## Data Availability

The datasets used and/or analyzed during the current study are available from the corresponding author upon reasonable request.

## Abbreviations

BIPs: Breakthrough infected patients
WT: Wild type
COVID-19: Coronavirus Disease 2019
SARS-CoV-2: Severe acute respiratory syndrome coronavirus 2
ACE2: Angiotensin-converting enzyme 2
AS: Ancestral strain
NAbs: Neutralizing antibodies
ORF: Open read frame
TNF-α: Tumor necrosis factor-α
IL: Interleukin
CI: Confidence interval
GMTs: Geometric mean titers
AEC: 3-Amino-9-ethylcarbazole
ELISPOT: Enzyme-linked immunospot
ID50: 50% Inhibitory dilution
NC: Negative control
SFU: Spot-forming units
RBD: Receptor binding domain
ELISA: Enzyme-linked immunosorbent assay
HRP: Horse radish peroxidase
PBMCs: Peripheral blood mononuclear cells
MBC: Memory B cells
