# Supplementary material for: Clinical characteristics and host immunity responses of SARS-CoV-2 Omicron variant BA.2 with deletion of ORF7a, ORF7b and ORF8
Source: Virol J. 2023 May 29;20:106. doi: 10.1186/s12985-023-02066-3 (PMC10226014; doi:10.1186/s12985-023-02066-3)
Supplement: Supplementary file 1 — Additional file 1. Primer pairs used for amplification and Sanger sequencing of detected region. [file 12985_2023_2066_MOESM1_ESM.docx]

Supplemental table1. Primer pairs used for amplification and Sanger sequencing of detected region.

| Primer | Sequence | Expected length |
| --- | --- | --- |
| F0 | 5' CTTCGTGGACATCTTCGTATTG 3' | 1593 |
| R0 | 5' TGGTAGCTCTTCGGTAGTAGCC 3' |  |
| F1 | 5' CTTCGCAGCGTGTAGCAGGTGA 3' | 1369 |
| R1 | 5' AGCGGTGAACCAAGACGCAGTA 3' |  |
